# Comparative analgesic efficacy of buprenorphine or clonidine with bupivacaine in the caesarean section

**DOI:** 10.4103/0019-5049.71046

**Published:** 2010

**Authors:** Kiran Agarwal, Navneet Agarwal, Vijender Agrawal, Ashok Agarwal, Mahender Sharma, Kanupriya Agarwal

**Affiliations:** Department of Obstetrics and Gynecology, Rohilkhand Medical College, Bareilly, Uttar Pradesh, India; 1 Department of Anesthesia, Rohilkhand Medical College, Bareilly, Uttar Pradesh, India; 2 Department of Community Medicine, Rohilkhand Medical College, Bareilly, Uttar Pradesh, India; 3 Department of Pediatrics, Rohilkhand Medical College, Bareilly, Uttar Pradesh, India; 4 Department of Obstetrics & Gynecology, Rohilkhand Medical College, Bareilly, Uttar Pradesh, India

**Keywords:** Bupivacaine, buprenorphine, caesarean section, clonidine, epidural analgesia

## Abstract

The need for early ambulation for caring of the neonate by mothers makes postoperative pain management after cesarean delivery unique. Favorable results have been observed with buprenorphine, clonidine and bupivacaine as epidural analgesics. This prospective, randomised triple blind control study was carried out among 112 lower segment caesarean segment (LSCS) patients, divided into three groups, to assess the analgesic efficacy and side effects of epidural analgesia, with an intermittent top up of (i) bupivacaine (0.125%) and buprenorphine (0.075 mg) (ii) bupivacaine (0.125%) and clonidine (37.5 microgram) and (iii) bupivacaine (0.125%) alone, in LSCS cases. The demographic characteristics (age, weight and height) of the three groups were comparable and the differences were not statistically significant. The mean duration of the analgesia was significantly longer in the group one patients receiving buprenorphine plus bupivacaine (690 ± 35 minutes) and it was lowest in group three patients receiving bupivacaine (170 ± 31 minutes) alone. The mean highest pain score (VAS scale) was significantly lower (3.4 ± 0.6) in group one patients and it was highest in group three (6.7 ± 0.8) patients. Requirement of continuation of epidural analgesia after 15 hours of operation and requirement of diclonfenac injections as well as incidence of itching and pruritus was significantly lower in group one patients. Incidence of nausea and vomiting was the lowest in group one patients. Incidence of respiratory depression, sedation and hypotension were nil in all three group of patients. Epidural buprenorphine combined with bupivacaine produced significantly longer duration and better quality of analgesia than bupivacaine combined with clonidine or bupivacaine alone, and it was safe in LSCS patients, for post-operative analgesia.

## INTRODUCTION

The need for early ambulation for caring of the neonate, by mothers, makes postoperative pain management after caesarean delivery unique. To achieve this, various drug combinations and techniques have been tried, to find out the more effective and safer analgesia. Favourable results have been observed with buprenorphine as an analgesic.[1,2] Buprenorphine is a mixed agonist — an antagonist narcotic with high affinity at both Mu (μ) and kappa (k) opiate receptors. It is an effective analgesic, similar to morphine, in nearly all-clinical situations. Buprenorphine is compatible with the cerebrospinal fluid (CSF) and produces no adverse reactions when administered intrathecally. Central neuraxial opioids, intrathecal as well as epidural, offer the perceived benefit of selective analgesia without sensory or motor blockade. However, side effects such as potentially catastrophic, delayed respiratory depression have prompted further research to develop non-opioid analgesics, with less worrisome side effects.[3] Intrathecal clonidine is being extensively evaluated as an alternative to neuraxial opioids for control of pain and has proven to be a potent analgesic, free of at least some of the opioid-related side effects.[4,5] It has been used as a sole agent as well as admixed with opioids and local anaesthetics, in labour analgesia and orthopaedic surgery. This prospective, triple-blind, randomised, controlled study was designed to assess the analgesic efficacy and side effects of epidural analgesia, with an intermittent top up of (i) bupivacaine (0.125%) and buprenorphine (0.075 mg) (ii) bupivacaine (0.125%) and clonidine (37.5 micrograms) and (iii) bupivacaine (0.125%) alone, for postoperative analgesia in lower segment caesarean section cases.

## METHODS

This study was conducted in a hospital of Uttar Pradesh (UP). It was designed in the form of a prospective, randomized and triple-blinded study. Approval for the study was obtained from the institutional ethical committee and a written informed consent from each patient was taken before the study. The postoperative Visual Analogue Scale (VAS) score was considered to be the primary end point in the determination of sample size. It was determined that a sample size of 36 patients in each group would have 80% power to detect 30% difference in VAS, while limiting the type I error to less than 5%. One hundred and twelve patients at term (ASA-I and ASA-II), scheduled for lower segment caesarean section under epidural anaesthesia, were selected by the simple random sample technique and divided into three groups. Patients with complicated pregnancy, acute foetal distress and history of hypersensitivity to opioids / local anaesthetics were excluded. In the pre-anaesthetic visit, all the patients were made familiar with the study plan and the different visual analogue scales (VAS) to be used in the assessment by the investigators. Respiratory rate, arterial blood pressure, peripheral arterial saturation and heart rate were monitored throughout the peri-operative period.

Epidural anaesthesia was used for surgery. Subsequently, the epidural route was used for postoperative analgesia also. Intensity of postoperative pain during the first 24 hours postoperatively was assessed at hourly intervals using a visual analogue pain score; 0 denoted ‘no pain’ while 10 denoted ‘worst pain imaginable’. When the patients were asleep, no VAS assessments were made and a VAS of 0 was given. Group one was scheduled to receive bupivacaine (0.125%) at three-hour intervals and buprenorphine (0.075 mg) at 12-hour intervals or when the pain score was 4 or more, or if the patient requested analgesia (whichever occurred earlier). Group two was scheduled to receive bupivacaine (0.125%) and clonidine (37.5 microgram) at three-hour intervals or when the pain score was 4 or more, or if the patient requested analgesia (whichever occurred earlier). Group three received bupivacaine (0.125%) alone at three-hour intervals or when the pain score was 4 or more, or if the patient requested analgesia (whichever occurred earlier). The observer assessing pain was kept blinded for the epidural medication. If the VAS score failed to decline at least by the first one, even after 30 minutes of epidural injection, the patient was given an injection of diclofenac sodium 75 mg intramuscularly. Total analgesics required for a 24-hour period was recorded.

The onset of analgesia was defined as the time from injection of the study medication to the first reduction in pain intensity by at least 1 in VAS; and the duration of analgesia was defined as the time between the onset of analgesia and either a return to baseline VAS or the time when additional pain medication was requested, whichever occurred first. The occurrence of nausea and vomiting, pruritus, shivering and respiratory depression (respiratory rate < 12 / minute), and sedation and hypotension was noted for up to 24 hours following administration of the study medication. The collected data was analyzed using the Statistical Package for Social Science (version 10.0 for Windows, SPSS). Analysis of variance / Chi square test was used to compare the variables between groups. A *P* value of < 0.05 was considered significant. The observer analyzing the data was kept blind about the groups to avoid bias in analysis and results.

## RESULTS

A total of 112 patients were studied. Group one and two consisted of 38 patients in each group, while group three consisted of 36 patients. The demographic characteristics (age, weight and height) of the three groups as mean, standard deviation and range are depicted in Table 1. The demographic characteristics of the three groups were comparable and differences among them were not statistically significant.

**Table 1:** Demograhic characteristics of patients

Characteristics	Group 1 (N = 38)	Group 2 (N = 38)	Group 3 (N = 36)	*P* value
Age in years {Mean ± SD (Range)}	27.21 ± 3.90 (20 – 37)	26.34 ± 4.06 (20 – 37)	26.82 ± 3.7 (18 – 36)	N S
Weight in kg {Mean ± SD (Range)}	69.12 ± 12.42 (49 – 112)	66.43 ± 10.64 (45 – 97)	62.96 ± 11.47 (46 – 82)	N S
Height in cm {Mean ± SD (Range)}	152.2 ± 1.2 (135 – 171)	153.5 ± 1.4 (135 – 174)	152.9 ± 1.8 (144 – 167)	N S

N S is not significant

**Figure 1:**
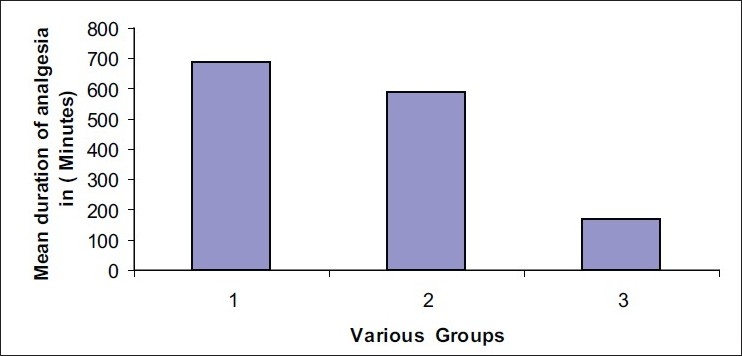
Mean duration of analgesia in various groups

Table 2 gives the comparison of post-operative analgesia in the three groups of patients. Mean duration of analgesia was significantly longer in group one patients receiving buprenorphine plus bupivacaine in comparison to group two patients receiving bupivacaine plus clonidine and it was the lowest in group three patients receiving bupivacaine alone [Figure 1]. The mean highest pain score (VAS scale) was significantly lower (3.4 ± 0.6) in group one patients and it was the highest in group three (6.7 ± 0.8) patients. Only 34% of the patients in group one required continuation of epidural analgesia after 15 hours, in comparison to 53% in group 2 and 76% in group three. Only 18.42% in group one required diclofenac injection in addition to epidural analgesia in comparison to 26.93% in group 2 and 73% in group three. These differences in the three groups were found to be statistically significant. The mean number of diclofenac injections required in group one was 0.18, in group two was 0.3 and in group three it was 1.35.

**Table 2:** Comparison of post-operative analgesia in the three groups

Effect	Group 1(N = 38)	Group 2 (N = 38)	Group 3 (N = 36)	*P* value
Duration of analgesia (minutes)	690 ± 35	590 ± 42	170 ± 31	< 0.05
Mean ± SD (Range)	(465 – 1290)	(455 – 1040)	(120 – 280)	significant
Highest pain score on VAS scale (0 – 10)	3.4 ± 0.6	4.5 ± 0.8	6.7 ± 0.8	< 0.05
Mean ± SD				significant
Percentage of patients requiring continuation of	34	53	76	< 0.05
epidural analgesia after 15 hours				significant
Percentage of patients requiring diclofenac injections	18.42	26.93	73	< 0.05
				significant
Number of diclofenac injection	0.18	0.3	1.35	N S
Mean (Range)	(0 – 1)	(0 – 2)	(1 – 3)	

N S is not significant

Table 3 depicts the comparison of the side effects of drugs in three groups of patients. Itching and pruritus were significantly higher in group three patients receiving bupivacaine alone and it was the lowest in group one receiving buprenorphine plus bupivacaine. Incidence of nausea and vomiting was the highest in group three patients and lowest in group two patients, but the differences were not found to be significant. Incidence of respiratory depression, sedation and hypotension were nil in all three group of patients.

**Table 3:** Comparison of side effects in three groups of patients

Side effect(Percentage)	Group 1(N = 38)	Group 2(N = 38)	Group 3(N = 36)	*P* value
Nausea	7	5	17	> 0.05 NS
Vomiting	5	3	11	> 0.05 NS
Itching	3	4	17	< 0.05 S
Pruritus	2	3	13	< 0.05 S
Respiratory depression	0	0	0	-
Sedation	0	0	0	-
Hypotension	0	0	0	-

NS: Not Significant, S: Significant

## DISCUSSION

This study has demonstrated that the lower doses of buprenorphine, in addition to bupivacaine, produce a longer duration of analgesia along with lower pain score and lesser requirement of diclonfenac injection in the post-operative period. It prolongs the duration of effective analgesia by a factor of three. Our finding has once again confirmed that a combination of an opioid and a local anaesthetic enhances the onset and prolongs the duration of analgesia more than the opioid or the local anaesthetic alone.[6] Abboud and coworkers, using higher doses of buprenorphine (1 to 4 mg) alone, epidurally, observed complete pain relief in 22 ± 2.4 minutes, but a remarkably longer duration of post-operative analgesia than that of our combination group.[7] Investigators have demonstrated that by using buprenorphine alone epidurally, in doses of 1 to 4 mg, varying durations of pain relief ranging from 2.5 to 9 hours are observed. As one can expect, with the use of lower doses of buprenorphine, the duration of pain relief in our study falls on the lower side of the reported range.[8]

Our study has demonstrated that buprenorphine produces a better quality of analgesia in terms of the VAS scale. Abboud and coworkers also found a significantly better quality of analgesia in patients receiving epidural bupivacaine with buprenorphine, rather than with bupivacaine alone. A recent study has shown that 2 mg of epidural buprenorphine added to a lower concentration of bupivacaine (0.1%) provided a better quality of labour analgesia in terms of incidence of motor blockade than 0.25% bupivacaine alone.[9] Furthermore, investigators have also found that analgesia provided by buprenorphine has a significant correlation with the affective domain, with greater reduction in affective magnitude than in pain intensity.[10] The incidence of sedation has been nil in all the three groups in our study. In contrast to our findings, a very high incidence of sedation has been reported with higher doses of epidural buprenorphine in post-caesarean patients.[11]

In our study, we did not observe itching, pruritus, or respiratory depression in any patient. Other studies of epidural buprenorphine for post-caesarean analgesia were also reported, with either a complete absence or a very low incidence of such side effects. In fact, the prophylactic administration of buprenorphine was recommended for the prevention of such side effects produced by pure agonist opioids such as morphine, and it was also effectively used for the treatment of intractable pruritus associated with dermatological conditions.[12,13] In our study the incidence of nausea and vomiting was lower in the buprenorphine group than in the bupivacaine group, but the differences were not found to be significant. A lower incidence of nausea and vomiting in the buprenorphine group could be due to lower doses of buprenorphine in comparison of other studies.

Various studies have demonstrated that addition of intrathecal clonidine to bupivacaine, even in very small doses, significantly improves the onset and duration of sensory and motor block, with relative haemodynamic stability.[14–16] In our study also the addition of clonidine to bupivacaine improved the onset and duration of analgesia in comparison to bupivacaine alone, but the duration of analgesia was significantly lower in comparison to buprenorphine combined with bupivacaine. The rapid onset and short duration of analgesia and lack of side effects with lower doses of epidural buprenorphine combined with bupivacaine, in our study, suggests that the drug may be particularly useful in situations where a prompt onset and / or limited duration of analgesia is indicated, patient-controlled epidural analgesia is used and side effects such as sedation are not desirable.

## CONCLUSION

Lower doses of epidural buprenorphine combined with bupivacaine produce a significantly earlier onset, longer duration and better quality of analgesia than bupivacaine combined with clonidine or bupivacaine alone, and is safe in lower segment caesarean section patients for post-operative analgesia.
